# How does accelerometry-measured arm elevation at work influence prospective risk of long-term sickness absence?

**DOI:** 10.5271/sjweh.4000

**Published:** 2022-02-25

**Authors:** by Nidhi Gupta, Charlotte Lund Rasmussen, Mikael Forsman, Karen Søgaard, Andreas Holtermann

**Affiliations:** 1 Department of musculoskeletal disorders and physical workload, National Research Centre for the Working Environment, Copenhagen, Denmark; 2 School of Engineering Sciences in Chemistry, Biotechnology and Health, KTH Royal Institute of Technology, Huddinge, Sweden;; 3 IMM Institute of Environmental Medicine, Karolinska Institutet, Stockholm, Sweden;; 4 Department of Sports Science and Clinical Biomechanics, University of Southern Denmark, Odense, Denmark;; 5 Department of Clinical Research, University of Southern Denmark, Odense, Denmark

**Keywords:** compositional data analysis, dose-response, elevated arm work, prevention

## Abstract

**Objective:**

Elevated arm work is prevalent in many jobs. Feasible device-based methods are available to measure elevated arm work. However, we lack knowledge on the association between device-measured elevated arm work and prospective risk of long-term sickness absence (LTSA). We aimed to investigate this association.

**Methods:**

At baseline, 937 workers wore accelerometers on the right arm and thigh over 1–5 workdays to measure work time spent with elevated arms in an upright position. Between baseline and 4-year prospective follow-up in the national registers, we obtained information on the individuals’ first event of LTSA (≥6 consecutive weeks). We performed compositional Cox proportional hazard analyses to model the association between work time with arm elevation >30°, >60°, or >90° and the probability of LTSA.

**Results:**

Workers spent 21% of their work time with >30° arm elevation, 4% with >60° arm elevation, and 1% with >90° arm elevation; in the upright body position. We found a positive dose–response association between work time spent with elevated arm work and the risk of LTSA. Specifically, we found that increasing two minutes of work time spent with arm elevation at (i) >90° increased the risk of LTSA by 14% [hazard ratio (HR) 1.14, 95% confidence intervals (95% CI 1.04–1.25)] (ii) >60° increased the LTSA risk by 3% (HR 1.03, 95% CI 1.03–1.06), and (iii) >30° increased the LTSA risk by 1% (HR 1.01, 95% CI 1.00–1.02).

**Conclusion:**

Device-measured elevated arm work is associated with increased prospective LTSA. This information ought to be brought into preventive workplace practice by accessible and feasible device-based methods of elevated arm work.

Sickness absence puts a large burden on employees, workplaces, and our society. In Denmark alone, 16 million sick days were reported in 2019, with a societal cost of almost 6 billion DKK (1).

A considerable fraction of the long-term sickness absence (LTSA) is associated with high physical work demands (2, 3). Work with elevated arms is prevalent in many jobs (4, 5). Elevated arm work puts a biomechanical load and restricted blood supply to shoulder and arm tissues (6). Thus, elevated arm work can lead to excessive fatigue, musculoskeletal pain, and LTSA (7–9).

Elevated arm work above shoulder level (or >90°) is associated with a higher risk of musculoskeletal pain (8, 10) while few studies have investigated the association with sickness absence (3, 7, 11). Additionally, a number of laboratory studies have shown the influence of time spent on arm elevation of various degrees (30°, 60° or 90°) on muscle fatigue and pain (12, 13). However, we lack such knowledge from studies on the arm elevation of various degrees during normal daily work and risk of sickness absence. Moreover, most knowledge on this topic is based on self-reported work time spent with elevated arms. Because it is very difficult to remember and estimate the amount of work time spent with elevated arms over the day, such information using self-reports is shown to be imprecise and potentially biased (14, 15). One study used an expert-rated job-exposure matrix and found that jobs with high mechanical exposures at work (including arm elevation >90° for >45 minutes per workday) were associated with increased sickness absence risk (16). However, the job-exposure matrices provide very gross estimate of exposure measures (17). This is because it is based on subjective rating provided by expert and that all workers within one job group get one rating of exposure that can vary substantially within a job group. These limitations can obscure the true relation between elevated arm work and risk of LTSA. Another option for measuring elevated arm work is via workplace visual observations (18). However, workplace observations are both costly and shown to be of rather low reliability (19). Thus, better methods to measured elevated arm work are needed to understand its true relation with risk of LTSA.

Freely available, accessible, and feasible device-based methods have recently been developed for accurate measurements of elevated arm work (20). However, because no study has investigated the association between device-measured elevated arm work and prospective register-based LTSA, we lack the required knowledge to interpret, evaluate and take workplace actions on preventing prevalent elevated arm work (21). This lack of knowledge hinders the use of feasible device-measured elevated arm work in workplace risk assessments and prevention of LTSA due to prevalent elevated arm work.

Thus, the study aimed to investigate the association between device-measured elevated arm work and prospective register-based LTSA.

## Methods

### Study design, population, and data

This is a prospective study with 4-years of follow-up time (212 weeks). Specifically, the study used baseline measurements on device-measured, elevated-arm work that were collected from workers enrolled in the cohort between 2011 to 2013. From the date of baseline measurement of each worker, the worker was followed for 212 weeks (approximately four years) in the national Danish registers to obtain information on the first event of an eventual long-term sickness absence (LTSA). This meant that workers (i) had an equal time of follow-up, exactly 212 weeks and (ii) ended the four-year follow-up on different dates between 2015 to 2017.

Baseline data comes from the ’technically measured compositional Physical wOrk DEmands and Prospective register-based Sickness Absence study (PODESA) cohort’ (2, 22). The PODESA cohort contains harmonized data from the NOMAD (23) and DPHACTO (24) cohorts. These two cohorts were easily harmonized due to similar procedures used to perform accelerometry for 1–5 working days and to collect additional information. Additionally, for participants in both of these cohorts, it was possible to access sickness absence data from national registers. More details on how we harmonized these cohorts and their background information are provided in our previously published protocol article (22).

The Ethics Committee for the Capital Region of Denmark approved the DPhacto and NOMAD cohorts (file number H-2-2012-011 and H-2-2011-047) (22). All eligible workers received written and oral information about (i) the practicalities of participation, (ii) potential risks of participating, and (iii) freedom of withdrawing from the project. Individuals gave a written consent to participate in the study and use their data for research studies. According to Danish law, questionnaire- and register-based studies do not need approval by ethical and scientific committees or informed consent (25).

With assistance from trade unions, we recruited a convenience sample of workers from 22 Danish workplaces in manufacturing, cleaning, transport, healthcare, garbage collection, construction, assembling, and mobile plant operations sectors in Denmark.

### Accelerometry

The workers wore two ActiGraph accelerometers (GT3X+, Florida, USA); one on the right thigh and one on the right upper arm for 1–5 consecutive working days (26). During these measurement days, the workers were also asked to complete a short diary indicating the time of starting and ending work, and the time of going to and getting out of bed each day.

After the measurement period, the workers returned the accelerometers to the research team. The accelerometer data were downloaded using Actilife Software version 5.5 and further processed using the MATLAB program Acti4 (27, 28). Acti4 uses a posture- (including arm elevation) and movement-identification algorithm that has previously been shown to have high sensitivity and specificity (27, 29). Using Acti4, thigh-based accelerometer and the self-reported diary information, we determined work time spent in upright position (ie, time spent standing, walking, running and stair climbing) and non-upright position (ie, seated). Additionally, combining the data from thigh-based accelerometer with data from arm accelerometer, we determined work time spent with arm elevation at ≤30°, >30°, ≤60°, >60°, ≤90° and >90° in upright position (26).

Based on the information in the self-reported diary, data were classified as work periods with hours spent in the primary occupation.

For the analysis, data for each participant on all postures and movements and total measured work time were averaged across all valid measured work periods (average/day). A work period was considered valid if it consisted of four hours or 75% of the average measured work time across days for a worker. In the analyses, we included all workers with at least one valid work period.

### Prospective register-based long-term sickness absence

The information on the first event of LTSA during the four years follow-up was retrieved from the Register-based Evaluation of Marginalization (DREAM) (30) using the workers’ unique civil registration number. The DREAM register contains information on granted subsidized sickness absence per week for each individual in Denmark. The sickness absence benefit is given by the state to the sick worker after 30 continuous days of sickness absence. LTSA event was defined as the first LTSA event that lasted for ≥6 consecutive weeks during the 4-year follow-up period from the last day of baseline measurements (31).

### Potential confounders

The potential confounders were chosen *a priori* based on the previous studies on the association between arm elevation at work and musculoskeletal pain and sickness absence (7–9; and supplementary material www.sjweh.fi/article/4000: Directed Acyclic Graph in Appendix G to justify the inclusion of selected confounders in the study). Information on the chosen confounders was collected at baseline via a questionnaire, a health check, or via the DREAM register. Age of the workers was determined using their unique civil registration number. Sex of the workers was determined using a single item ’are you male or female?’ Body mass index (BMI) of the workers was determined using objective measurements of weight (kg) and height (cm). Work time spent with lifting and carrying was determined using a single item with six responses ranging from ’almost all the time’ to ’never’ (32). Information on ’type of work’ was determined using a single item ’are you a worker engaged in administrative work tasks (white-collar) or production (blue-collar)? Information on the event of LTSA within 12 months before baseline was determined using DREAM register. Information on the influence at work was determined using two items from the Copenhagen Psychosocial Questionnaire (33). The responses were summarized and translated into a scale of 0–100% where 0 meant no influence at work.

### Statistical analysis

All analyses were performed using a compositional data analysis (CoDA) approach. For a more detailed description of the implementation of CoDA in occupational research, please read reference (34). Our exposure was work time spent with arm elevation which is compositional data because of two properties: (i) if work time spent with arm elevation is increased or decreased, it will modify work time spent on at least one other exposure and (ii) data on work time spent with elevated arm are constrained in nature as it can only range between 0–100% work time. Traditional statistical methods are not suitable to handle compositional data. Instead, we are recommended to use CoDA-based methods. In CoDA, the first step is to transform the data from the constrained data space (ie, compositional data space) to a space where data can range freely between -∞ to +∞. The most popular transformation method has been the isometric log-ratios (ilrs) that results in D-1 log-ratios where D is number of parts within a composition. Thereafter, any standard statistical methods can be used to analyze the transformed data. However, the interpretation of the resulting estimates based on the transformed data can be difficult. Thus, the analyses are usually coupled with predictions methods helping to interpret the resulting estimates.

**Figure 1:**
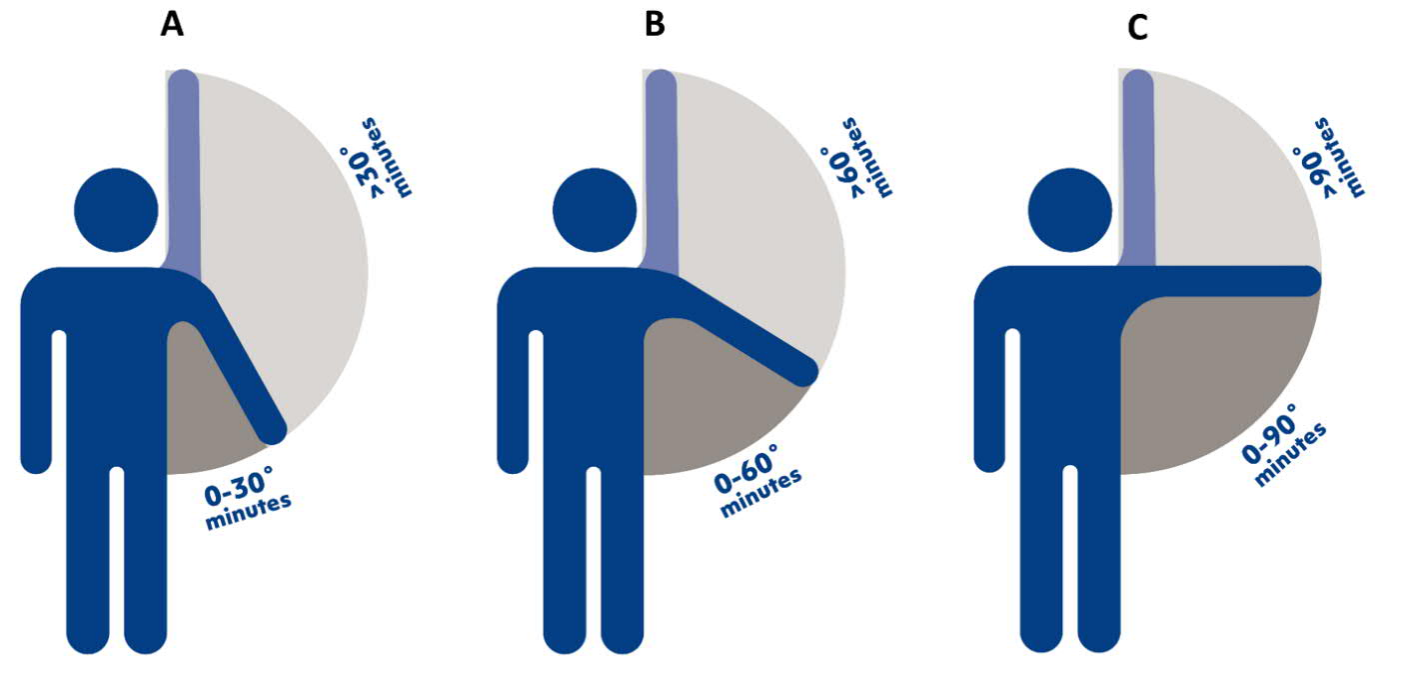
Definition of the three worktime compositions of arm elevation while in an upright position that were used in analyzing the association between relative work time spent with arm elevation at >30° (A), >60° (B), and >90° (C) and risk of long-term sickness absence.

We performed three sets of CoDA-based analyses on exposures of interest of the study: three compositions of work time (figure 1). Each composition contained three parts: (i) “Composition A”: arm elevation >30° in upright body position, arm elevation ≤30° in upright body position, and total non-upright body position or seated position (figure 1A); (ii) “Composition B”: Arm elevation >60° in upright body position, arm elevation ≤60° in upright body position, and total non-upright body position (figure 1B), and (iii) “Composition C”: Arm elevation >90° in upright body position, arm elevation ≤90° in upright body position, and total non-upright body position (figure 1C).

In all compositions, we focused on arm elevation during upright position only and not during non-upright position. Although we did not have contextual information on when arms were supported at work, we believe the arms are more supported in non-upright position (seated position) compared to upright position. When arms are supported, the elevated arm work does not increase the load or strain on the shoulders (5, 26), thus elevated arm work with arm support does not pose a health risk to the workers. Please refer to sensitivity analysis 1 if considering the arm elevation while in only upright position as in the main analyses instead of considering the arm elevation during both upright and non-upright position could give different results.

### Main analyses

The main CoDA analyses consisted of three steps.

*Step 1*. We transformed each work time composition to ilrs [see (35) to understand how ilrs are calculated]. For each composition, this transformation resulted in two ilrs (ilr_1_ and ilr_2_). Ilr_1_ indicates the ratio of time spent with arm elevation of a higher degree in upright position relative to time spent with arm elevation of a lower degree in upright position and total time spent in a non-upright position. Ilr_2_ indicates the ratio of time spent with arm elevation of a lower degree in upright position and total time spent in non-upright position. The equations for calculating these ilrs are given in the Appendix A.

*Step 2*. We performed three separate Cox proportional hazards regression analyses (respectively for each composition), modeling both ilrs (as continuous variables) against the first event of LTSA. The models were first adjusted for age and sex only (crude model). In the fully adjusted models, we also adjusted for BMI, work time spent with lifting/carrying, and type of work (blue- or white-collar). The resulting estimates from for the Cox models (both crude and fully-adjusted) on each composition are given in supplementary appendix B.

In the Cox models, each worker contributed with risk time until the first event of LTSA occurred or until the end of a 4-year follow-up in case of no event. A total of 45 workers were censored – ie, dropped out during the 4-year follow-up due to one of the following reasons: emigrated, died, entered early retirement, entered ordinary retirement, or became pregnant (ie, going on maternity leave 8 months following baseline). These censored workers contributed to the risk time in the analyses until the week of dropping out.

The assumption of the proportional hazards was tested via visual inspection and via the Grambsch-Therneau test (36). The statistical significance of the association between work time compositions and LTSA risk was assessed using the Type-II likelihood-ratio tests. The results were considered significant at P<0.05.

**Figure 2:**
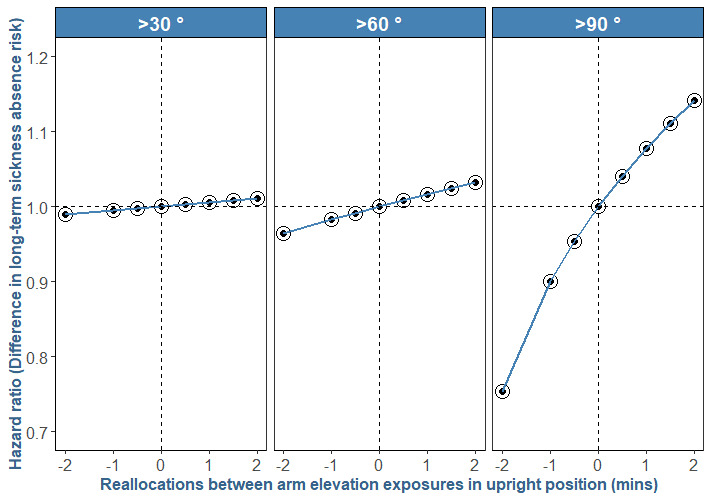
Results of the direction and strength of the association between work time spent with arm elevation >30°, >60° and >90° in upright position, relative to work time spent with arm elevation ≤30°, ≤60°, and ≤90°, respectively, and prospective risk of long-term sickness absence. The X-axis represents reallocations of up to 2 minutes between; composition A: >30° and ≤30°, composition B: >60° and ≤60°, and composition C: >90° and ≤90° in upright position. Y-axis indicates the ratio of the hazards associated with the new reallocated composition and reference composition (average composition). “0” on y axis represents risk associated with reference average composition. The displayed association looks non-linear for panel “>90°”. This is because when linear equations are performed on ilrs (the transformed composition A, B or C) and the results are then anti-logged, the results appear to be non-linear. A detailed explanation for why this happens is given in reference (46).

*Step 3*. To interpret the resulting ilr-based estimates (reported in supplementary appendix B) obtained from each Cox analysis, we implemented a previously used analysis (32, 37) that is described in supplementary appendix A with one detailed example. First, the reference was determined that is the “average composition” A, B, or C as shown in figure 1. Second, from this reference composition, new theoretical compositions of work time were determined by reallocating a fixed amount of time from one part to another part, so that the total average composition time is kept constant. We performed these reallocations with increments of ±2 minutes from the average composition, resulting into nine new theoretical compositions that we transformed into ilrs. Third, using the Cox-estimates from step 2, we predicted hazard ratios (HR) and their 95% confidence intervals (CI) indicating the predicted difference in LTSA risk corresponding to the difference in new theoretical composition and the “reference composition”. The formula for calculating these predicted HR and their 95% CI are given in supplementary appendix A. Finally, we plotted these predicted HR and their 95% CI against corresponding reallocations (±2 minutes) as shown in figure 2.

We chose such small reallocations of ±2 minutes because we wanted to compare the results of all three compositions A, B and C (figure 1), which required the increments to be of similar duration for all three compositions. We chose ±2 minutes reallocations because of a small average (3 minutes) and a narrow range (5–95^th^ percentile: 0.5–13 minutes) for time spent with arm elevation >90° (composition C). This also meant that we performed the reallocations that produced theoretical compositions covering a wide range of composition C but only a ’partial’ range for composition A and B.

We repeated step 3 on a new set of theoretical compositions (called “second-set compositions” shown in supplementary appendix C). For these second-set compositions, instead of the average composition, the reference composition contained the minimum exposure to arm elevation of higher degrees in each composition. The reallocation increments were large enough to cover the whole range (5–95^th^ percentile) of work time spent with arm elevation of higher degrees. The range of these reallocations was for Composition A: 24–169 minutes, Composition B: 3–46 minutes and Composition C: 0.5–13 minutes. The predicted HR indicated how the risk of LTSA developed over the whole range of work time spent with arm elevation. We plotted the predicted HR against the measured 5–95^th^ percentile range (in minutes) of arm elevation of higher degrees for each composition as shown in supplementary appendix E.

### Sensitivity analyses

To test the sensitivity of the results obtained from the main analyses, we also performed three sensitivity analyses: (i) For the primary analyses, we split the arm elevation information in upright position but not during the non-upright position. However, we wanted to see if the findings remained robust when not splitting the arm elevation time in upright and non-upright body position. Thus, we investigated the association between the arm elevation during the whole work time – not differentiating between upright and non-upright position – and risk of LTSA; (ii) Due to technical errors, we did not obtain answers from 204 workers for the questions on influence at work. Thus, the main analyses were performed without and with additional adjustment for influence at work on the remaining 733 workers; (iii) We also performed a separate analysis where we excluded the workers who had pre-events of LTSA, ie, events within 12 months before baseline (N=57).

All analyses were performed in the R software [version (3.5.1)] using the software packages ’robCompositions’ (38) and ’survival’ (39).

## Results

### Participants flow and descriptive

Of the invited 2498 eligible workers, in the main analyses, we included 937 workers with valid data on at least one working period (measured work time/day = 457 minutes) and with information on potential first event of LTSA from the national register. More details on the flow of the participants are given in supplementary appendix D.

These 937 workers were on average 45 years old and had a BMI of 27 kg/m^2^. Additionally, 44% of them were women, and 93% of them were engaged in blue-collar occupations (table 1). In total, 201 workers (21%) had their first event of LTSA at on average (median) 76^th^ week within the 4-year (ie, 212 weeks) follow-up time.

**Table 1:** Descriptive of the workers without (N=736) and with (N=201) an event of long-term sickness absence and the total workers (N=937). [LTSA=long-term sickness absence; SD=standard deviation]

Variables	Without event (N=736)			With event (N=201)			Total (N=937)		
		
	N	%	Mean (SD)	N	%	Mean (SD)	N	%	Mean (SD)
Age (years)	736		45.1 (9.7)	201		43.8 (10.3)	937		44.8 (9.8)
Women	299	41		113	56		412	44	
BMI (kg/m^2^)	723		27.2 (4.9)	199		27.1(5.2)	922		27.1 (4.9)
Occupational lifting/carrying duration (1–6) ^a^	733		3.8 (1.4)	201		3.5(1.5)	937		3.7 (1.4)
Influence at work (0–100%) ^b^	578		58.2 (28.2)	155		52.6(30.0)	733		57.0 (28.7)
White-collar	55	7		9	4		64	7	
Blue-collar	681	93		192	96		873	93	
Job sector									
Cleaning	117	16		46	23		163	17	
Manufacturing	441	60		110	55		551	59	
Transport	60	8		19	10		79	8	
Health Service	11	2		7	4		18	2	
Assemblers	29	4		3	2		32	3	
Construction	30	4		7	4		37	4	
Garbage Collectors	19	3		6	3		25	3	
Mobile Plant Operators and others ^c^	29	4		3	2		32	4	
Pre-event of LTSA	29	4		28	14		57	6	
Pregnant at baseline	23	3		0	0		23	3	
Total measured worktime/day			457 (91.4)			453 (81.8)			457 (89.4)
Wrist/hand pain (yes) ^d^	163	22		64	32		227	24	
Neck/shoulder pain (yes) ^d^	289	39		99	50		388	42	
Angina Pectoris (yes) ^e^	10	2		4	3		14	2	

a 1=almost all the time, 6=never.

b 0% meant no influence at work.

c General office clerks and other elementary workers.

d At baseline, the information on the wrist/hand and neck/shoulder pain was obtained by asking if participants had experienced pain in these regions in the past 7 days with yes/no responses.

e At baseline, the information on event of Angina Pectoris was obtained by asking if they had experienced this event previously.

Of the measured work time/day of 457 minutes, 155 minutes were spent in non-upright body position (sitting or lying) and remaining 302 minutes in the upright body position. Of the working time spent in the upright body position, workers spent; 94 minutes with >30° and remaining 208 minutes with arm elevation ≤30° (composition A); 17 minutes with >60° and remaining 285 min with arm elevation ≤60° (composition B); and 3 minutes with >90° and remaining 299 minutes with arm elevation ≤90° (composition C), respectively (table 2).

**Table 2:** Compositional means of the accelerometer-measured work time spent on non-upright position and arm elevation of various degrees while in upright position among workers without (N=736) and with (N=201) an event of long-term sickness absence ^a^ and the total workers (N=937).

Accelerometer-measured work time compositions (mean)	Without event (N=736) (minutes/day)	With event (N=201) (minutes/day)	Total workers (N=937) (minutes/day)
Total measured work time/day	457	453	457
Total measured work time/day in non-upright position	155	148	155
Total measured work time/day in upright position	302	305	302
Composition A ^b^			
Arm elevation >30° in upright body position	94	98	94
Arm elevation ≤30° in upright body position	208	207	208
Total non-upright position	155	148	155
Composition B ^b^			
Arm elevation >60° in upright body position	16	18	17
Arm elevation ≤60° in upright body position	286	287	285
Total non-upright position	155	148	155
Composition C ^b^			
Arm elevation >90° in upright body position	3	3	3
Arm elevation ≤90° in upright body position	299	302	299
Total non-upright position	155	148	155

a An event of long-term sickness absence was defined as the first event of long-term sickness absence that lasted for ≥6 consecutive weeks during the 4-year follow-up period from the last day of baseline.

b Each “composition” represents the 100% work time spent on three exposures in each composition, as shown in the table.

Comparing workers with (N=201) and without (N=736) LTSA event, no major differences in descriptive characteristics were found, except that the group of workers with LTSA event had relatively more women, had slightly less influence at work and had more pre-events of LTSA (ie, LTSA event during 12 months before baseline) (see table 1).

### Main analysis

Results of the compositional Cox proportional hazard models indicated that, based on the fully-adjusted models, there was a significant respective association between work time spent on composition A (P=0.04), composition B (P=0.05), and composition C (P=0.001) and risk of LTSA (composition A, B, and C shown in figure 1). Results were similar when models were adjusted for only age and sex and when further adjusting for age, sex, BMI, work time spent with lifting/carrying, and type of work (results shown in supplementary appendix B).

### Results on the first-set compositions

In composition A, reallocating two more minutes to arm elevation >30° from arm elevation ≤30°, keeping the remaining work time, ie, non-upright, constant, was associated with 1% (HR 1.01, 95% CI 1.00–1.02) higher risk of LTSA. In composition B, reallocating two more minutes to arm elevation >60° from ≤60° was associated with 3% (HR 1.03, 95% CI 1.01–1.06) higher LTSA risk. In composition C, two more minutes to arm elevation >90°from ≤90° was associated with 14% (≥90° HR 1.14, 95% CI 1.04–1.25) higher LTSA risk (figure 2).

### Results on the second-set compositions

In a separate analysis on the second set of work time compositions (supplementary appendix C), we determined how the LTSA risk develops over the entire range of elevated arm work at various degrees. We found that compared to null exposure, increasing 124 minutes time spent with arm elevation >30° (thus reducing 124 minutes from ≤30° and keeping 155 minutes with non-upright position constant) was associated with a two-fold higher risk of LTSA (supplementary appendices C and E). Such two-fold risk of LTSA were observed at increasing 37 minutes of work time with arm elevation >60° and increasing only 8 minutes of work time with arm elevation >90°.

### Sensitivity analyses

We performed three sensitivity analyses to test the robustness of the main results as shown in supplementary appendix F. Overall, we found that the results of the main analyses and these sensitivity analyses were similar.

## Discussion

We investigated the association between device-measured elevated arm work and prospective register-based LTSA risk. We found a clear positive dose–response association between work time spent with arm elevation and LTSA risk. Specifically, we found that this dose–response association was steeper at higher degrees of arm elevation.

To the best of our knowledge, this is the first study investigating the association between device-measured work time spent with arm elevation and LTSA risk. The results of a positive association between arm elevation at work and LTSA risk are in line with previous studies using self-reports or job exposure matrix to determine elevated arm work (7, 16, 40). However, the levels of work time spent with arm elevation in these previous studies were very high. For example, in a national survey in Denmark, 20% of the workers reported that on average they spent >25% of the work time with arm elevation >90° (4). However, in our study, none of the workers were exposed to such high levels of work time spent with arm elevation >90°. Thus, the findings of these previous studies based on self-reports- or job exposure matrix-based arm elevation are not comparable to our study. The previous studies using device-measured elevated arm work did not investigate the association with sickness absence risk (8, 41). Thus, there is a need for more studies on device-measured elevated arm work and prospective LTSA risk to test the validity and generalizability of our findings.

We also found that the positive dose–response association between time spent with elevated arm work and risk of LTSA was steeper at higher degrees of arm elevation (figure 1). This meant that increasing similar amount of work time of arm elevation, the LTSA risk was much higher at higher degrees of arm elevation than lower degrees of arm elevation. However, please note that workers generally spent higher work time with lower degrees arm elevation (eg, on average, 94 minutes with arm elevation >30°) than higher degrees arm elevation (eg, on average 17 minutes with arm elevation >60° and 3 minutes with arm elevation >90°; table 2). Thus, workers had to spend higher time with arm elevation of lower degrees to observe similar LTSA risk compared to time spent with arm elevation of higher degrees. These results are in line with the experimental studies that have shown that with increasing arm elevation degree, the intra-muscular pressure in the infra- and supraspinatus muscles increases (12). This accentuates hypovascularity and blood flow impairment in these muscles resulting into reduced recovery, higher fatigue and in the long-run musculoskeletal pain and sickness absence (13). However, due to lack of similar epidemiological studies, we could not compare our results with previous research. Thus, we require similar epidemiological studies using device-based measurements of elevated arm work to facilitate confirmation of our results.

### The practical implication of the results

First, we believe that our study is the first to provide knowledge to practitioners, workplaces, and policy-makers on precise, device-measured, elevated arm work and LTSA risks. This is because the exposure levels of elevated arm work from self-reported studies have been much higher, and unreliable in comparison to the actual workplace exposures to elevated arm work. Thus, the results of this study provide precise knowledge on an association between elevated arm work and LTSA risk.

Second, the observed effect sizes in our study were significant and of practical relevance. Two minutes of reallocation between arm elevation ≤30° and >30° was associated with 1% change in LTSA risk (figure 2). This effect size seems small because it corresponds to only 2% difference from the average exposure of arm elevation >30° (ie, 2 minutes reallocation is 2% of the average 94 minutes time spent on arm elevation >30°). If we choose a higher reallocation of time spent on arm elevation >30°, the effect sizes will look much larger as shown in appendices E and H. For example, appendix H shows that reallocating 20 minutes (being about 20% of the total work time spent on arm elevation>30°) between arm elevation ≤30° and >30° is associated with 11% change in LTSA risk. Thus, if it is feasible to change ≥20 minutes exposure of arm elevation >30° at work, the observed effect would indeed be of practical relevance for the prevention of LTSA.

Third, feasible and accessible device-based methods to measure elevated arm work are already developed and available for researchers and practitioners where this precise knowledge can be integrated (42). One example of such method is ErgoArmMeter (42) which uses an app on a phone attached on the arm to measure accurate time spent with the arm in various degrees of elevation. Direct postural feedback via smartphone apps and so-called smart textiles have also been positively tested, in short-term interventions (43). Very recently researchers have proposed thresholds for when upper arm work becomes a risk for musculoskeletal pain (44). However, these thresholds are not based on evidence from prospective studies and we still lack knowledge on the precise dose of work time spent with elevated arm work and the risk of LTSA. Currently, the available feasible and accessible methods cannot provide such knowledge to researchers and practitioners. Our results can provide relevant knowledge as to when specific minutes of exposure to elevated arm work increases LTSA risk. For instance, our results indicate that when exposure to arm elevation >60° increases from null to almost ten minutes, the risk of LTSA increases by approximately 50% (Appendix E). The integration of such knowledge into the feasible and accessible device-based method may help workplaces to determine the existing exposure to elevated arm work of various degrees and determine the current risk of LTSA due to time spent on elevated arm work among workers. Such accurate risk assessment may help workplaces to design realistic, data-driven, and evidence-based interventions to prevent harmful elevated arm work. Thus, the knowledge from the study will help to establish an accurate and easy risk assessment for better workplace prevention.

### Strengths, limitations, and methodological considerations

One limitation of the study is that only 37% of the total sample was included in the main analyses. This is because not all workers were offered to participate in arm accelerometry. Previous studies on these data have also shown no relevant differences in their demographics (eg, age, gender distribution) between participants and non-participants (24). Another limitation is a lack of information on the “load” (eg, if the arms were supported and if the workers were lifting any weight) while elevating arms, which limited us to elaborate on our findings. Another limitation is lack of contextual information on work tasks including elevated arm work. Future studies should strive to collect such supplementary information on load and contextual factors. Another limitation is that we lack cause-specific information on LTSA. As shown in table 1, the workers with LTSA had a higher prevalence of musculoskeletal pain compared to those without LTSA events. Thus, there is a high probability that the observed detrimental association between elevated arm work and LTSA may be related to LTSA specific to musculoskeletal pain. Cause-specific information on LTSA might have indicated if the observed LTSA risk was mainly due to musculoskeletal pain or due to some other diseases. On the other hand, because of the high co-morbidity between musculoskeletal disorders and other causes of LTSA, such as depression and anxiety (45), the validity of musculoskeletal pain-specific LTSA can be questioned. Thus, the absence of cause-specific LTSA may not be considered a major limitation of our study. Another limitation is the availability of information on only a few job groups that limit the generalizability of our results to other job groups. The limitation of the study is also the potential presence of residual confounding that can always occur in observational non-randomized studies.

One strength of the study is the device-measured information work time spent with arm elevation of various degrees. Another strength is the usage of CoDA that is a recommended analytic method to handle compositional data like work time spent with arm elevation as in our study. Yet another strength is the prospective register-based LTSA.

In our main analyses, we chose to investigate the LTSA risk associated with arm elevation during upright position only, and not during non-upright position. To investigate this further, we performed a separate analysis investigating the association between arm elevation during the entire work time and LTSA. We observed similar trends as in the main results (Appendix F), but as expected, the associations between elevated arm work of various degrees during the entire work time and LTSA were weaker. For the results based on two minutes reallocations, the CI of the resulting HR look narrow. Had it been that the corresponding reallocation was larger (say 20 minutes reallocations), the 95% CI of the HR would have been wider. The reason for this is that the farther you move from the average value of a distribution (2 minutes reallocation to 20 minutes reallocation), the less confident we are about the estimated effect, thus producing wider CI (seen in appendix H).

### Concluding remarks

For the first time, a dose–response association between precise device-based measurements of elevated arm work and prospective risk of LTSA risk was observed. The dose–response association between elevated arm work and LTSA was steeper at higher degrees of arm elevation. Such knowledge can be brought into preventive workplace practice by accessible and feasible device-based methods of measuring elevated arm work.

## Supplementary material

Supplementary material
