# Supplementary material for: How does accelerometry-measured arm elevation at work influence prospective risk of long-term sickness absence?
Source: Scand J Work Environ Health. 2022 Feb 25;48(2):137–47. doi: 10.5271/sjweh.4000 (PMC9045232; doi:10.5271/sjweh.4000)
Supplement: Supplementary material [file SJWEH-48-137-S001.pdf]

How does accelerometry-measured arm elevation at work influence prospective risk of long-term sickness absence?[1]

by Nidhi Gupta, PhD,[2] Charlotte Lund Rasmussen, PhD, Mikael Forsman, PhD, Karen Søgaard, PhD, Andreas Holtermann, PhD

1. APPENDICES
2. Correspondence to: Nidhi Gupta, National Research Centre for the Working Environment, Lerso Parkalle 105, DK-2100 Copenhagen, Denmark. [E-mail: ngu@nfa.dk]

Appendix A. An example showing how we compare the traditional cox analytics and compositional cox analytics in investigating the association between the worktime composition B and risk of long-term sickness absence. The worktime composition B contains worktime spent with arm elevation >60˚ in upright body position, arm elevation ≤60˚ in upright body position, and total non-upright body position.

Analytical Steps	Traditional Cox analytics	Compositional Cox analytics
Exposure definition (continuous variables in minutes)	Minutes spent on three exposures (i.e., arm elevation>60˚, arm elevation ≤60˚, and non-upright position) treated as three continuous independent exposures.	These three exposures within a composition of worktime are transformed into isometric log-ratios (ilrs) resulting in two ilrs:<br>1. $ilr1_i = \sqrt{\frac{2}{3}}\ln\left(\frac{\text{Arm Elevation>}60^o\ (min)_i}{\sqrt[2]{Arm\ Elevation{\leq}60^o\ _{(min)i}{\times}Non{-}upright\ position(min)_i}}\right)$<br>2. $ilr2_i = \sqrt{\frac{1}{2}}\ln\left(\frac{\text{Arm Elevation}{\leq}60^o{}_i}{Non{-}upright\ position_i}\right)$<br>Where *i* is one worker<br>The unit of the exposure is irreverent to obtain ilrs.
Cox model	$h_{LTSA}(t) = h_0(t)\exp(B_1 arm\ elevation \leq 60^0 + B_2 arm\ elevation > 60^0 + B_3 non - upright\ position + B_4 age + B_5 sex + B_6 BMI + B_7 lift/carry + B_8 worktype)$<br><br>$ln\left(\frac{h_{LTSA}(t)}{h_0(t)}\right) = B_1 \leq 60^0 + B_2 > 60^0 + B_3 non - upright + B_4 age + B_5 sex + B_6 BMI + B_7 lift/carry + B_8 worktype$<br><br>Where<br>$H_0$=baseline hazard where the three exposures are not present<br>B_1-B_3=estimates of the three exposures	$h_{LTSA}(t) = h_0(t)\exp(B_1 ilr_1 + B_2 ilr_2 + B_4 age + B_5 sex + B_6 BMI + B_7 lift/carry + B_8 worktype)$<br><br>So<br>$ln\left(\frac{h_{LTSA}(t)}{h_0(t)}\right) = B_1 ilr_1 + B_2 ilr_2 + B_4 age + B_5 sex + B_6 BMI + B_7 lift/carry + B_8 worktype)$<br><br>Where<br>$H_0$=baseline hazard where exposures ("expressed as 2 ilrs") are not present<br>B_1-B_2=estimates of the two ilrs<br>B_4-B_8=estimates of the confounders

<table>
<tr><td></td><td>B_4-B_8=estimates of the confounders</td><td></td></tr>
<tr><td>Results of Cox estimates</td><td>
<table>
<tr><th>variables</th><th>HR and 95%CI</th></tr>
<tr><td>Arm elevation ≤60˚</td><td>0.999 (0.997—1.001)</td></tr>
<tr><td>Arm Elevation >60˚</td><td>1.003(0.998-1.008)</td></tr>
<tr><td>Total Non-upright position</td><td>1.0003 (0.998—1.002)</td></tr>
</table>
</td><td>
<table>
<tr><th>Variables</th><th>B (95%CI)</th><th>SE(B)</th><th>HR</th></tr>
<tr><td>Ilr$_1$</td><td>0.3227 (0.052—0.593)</td><td>0.138</td><td>1.38 (1.05—1.81)</td></tr>
<tr><td>Ilr$_2$</td><td>-0.353 (-0.638— -0.068)</td><td>0.145</td><td>0.70 (0.53—0.93)</td></tr>
</table>
</td></tr>
<tr><td>Interpretation of the Cox estimates</td><td>This analysis gives three HR estimates, one for each exposure (time spent with arm elevation >60˚in upright position, arm elevation ≤60˚ in upright position, and total time spent on non-upright position). For one of the exposures (i.e. time spent with arm elevation >60˚in upright position), the interpretation of the Cox HR estimates will be: per minute increase in time spent with arm elevation >60˚, the risk of LTSA increases by 0.3%.

Please note that these estimates are based on continuous exposures in “minutes” and not in “hours” (this is why the HRs might seem small with a narrow 95%CI).</td><td>This analysis gives two HR estimates instead of three like in traditional analytics. This is because, in the compositional analytics, the exposures are two isometric log-ratios instead of the three exposures (time spent with arm elevation >60˚in upright position, arm elevation ≤60˚ in upright position, and total time spent on non-upright position) like in the traditional analytics.

Example of how to interpret one of the ilr$_1$-based HR: One unit increase in a log of worktime spent with arm elevation >60˚ in upright position relative to arm elevation ≤60˚ in upright position and total non-upright position increases LTSA risk by 38%.

Because we cannot interpret what is ‘one unit of log’, it is difficult to interpret the HR estimates. Thus, we did the following: we first determined the sample average of Composition B (shown in bold in the table below). From this average composition, We then determined the new theoretical compositions by reallocating a fixed amount of time from one part to another part of the composition, so that the total average composition time is kept constant. This way, we determined nine theoretical compositions that are shown in the Table below.</td></tr>
</table>

Number of compositions	Reallocation (mins)	Arm elevation >60˚ (mins)	Arm elevation ≤60˚ (mins)	Non-upright (mins)	Ilr_1	Ilr_2
1	-2.0	14.7	287.3	155	-2.171	0.442
2	-1.5	15.2	286.8	155	-2.143	0.441
3	-1.0	15.7	286.3	155	-2.116	0.440
4	-0.5	16.2	285.8	155	-2.09	0.438
**Average composition**	**0**	**16.7**	**285.3**	**155**	**-2.065**	**0.437**
6	0.5	17.2	284.8	155	-2.04	0.436
7	1.0	17.7	284.3	155	-2.016	0.435
8	1.5	18.2	283.8	155	-1.992	0.433
9	2.0	18.7	283.3	155	-1.970	0.432

Using the cox model estimates (the two ilrs-HRs shown above), we predicted the hazard ratios that indicated what would be the LTSA risk if workers had one of the 9 new theoretical compositions compared to the sample average composition using the following formula:

$$HR = \exp\left[\hat{\beta}_1(x - \bar{x}) + \hat{\beta}_2(y - \bar{y})\right]$$

Where
HR = predicted hazard ratio
x and y = new theoretical ilrs (shown in the table above)
$\bar{x}$ and $\bar{y}$ = average ilrs (row 5 in the table above)
$\hat{\beta}_1$ and $\hat{\beta}_2$= parameter estimates for ilr_1 and ilr_2, respectively

We calculated the 95%CI of this HR using the following formula:

$$\exp\left[\hat{\beta}_1(x - \bar{x}) + \hat{\beta}_2(y - \bar{y}) \pm 1.96 \times \sqrt{(x - \bar{x})^2 Var(\hat{\beta}_1) + (y - \bar{y})^2 Var(\hat{\beta}_2) + 2 \times (x - \bar{x})(y - \bar{y})\, Cov(\hat{\beta}_1, \hat{\beta}_2)}\right]$$

where
Var=variance
Cov=covariance

We give below an example of how we calculated these HR and its 95%CI:

1. *Average composition calculation and corresponding ilrs*

	16.7 minutes arm elevation in upright position >60˚, 285.3 minutes arm elevation ≤60˚ in upright position and 155 minutes on non-upright position, translated to<br>Ilr_1 = -2.064506(x in formula above)<br>Ilr_2 = 0.4372112(y in formula above)<br><br>*2. New theoretical composition calculation using 2 minutes reallocation*<br>14.7 minutes arm elevation in upright position >60˚, 287.3 minutes arm elevation ≤60˚ in upright position and 155 minutes on non-upright position translated to<br>Average ilr_1 = -2.171144 ($\bar{x}$)<br>Average ilr_2 = 0.4421373 ($\bar{y}$)<br><br>*3. Calculate HR using the formula written above*<br>$HR = \exp[0.3227719(-2.171144 - -2.064506) + -0.3528727(0.4421373 - 0.4372112)]$ HR=0.964<br><br>*4. calculate 95%CI of the HR using the formula given above*<br>*Given that*<br>$cov(\hat{\beta}_1, \hat{\beta}_2)$=-0.01414071<br>Var $(\hat{\beta}_1) = 0.01906124$<br>Var$(\hat{\beta}_2)$= 0.02111989<br><br>$$\exp\left[0.3227719(-2.171144 - -2.064506) + -0.3528727(0.4421373 - 0.4372112) \pm 1.96 \times \sqrt{\begin{matrix}(-2.171144 - -2.064506)^2\ 0.01906124 + \\ (0.4421373 - 0.4372112)^2\ 0.02111989 + \\ 2 \times (-2.171144 - -2.064506)(0.4421373 - 0.4372112) - 0.01414071\end{matrix}}\right]$$<br><br>**Resulting 95%CI = 0.936-0.994**<br><br>The reason behind why we have such a narrow confidence interval of the HR is because this confidence interval corresponds to a very small difference of only 2 minutes between the new theoretical composition and the average composition ($-2.171144 - -2.064506$) = -0.107. Had it been the corresponding difference of 16 minutes, the 95%CI confidence interval of the HR would have been 0.22-0.87.

Appendix B. Resulting estimates of the Cox Proportional Hazard models to investigate the association between each composition (A, B, or C) of worktime spent on elevated arm work and risk of long-term sickness absence.

	Variables	B	SE(B)	P
Composition A: arm elevation >30˚ in upright body position, arm elevation ≤30˚ in upright body position, and total non-upright body position				
Crude	Ilr_1	0.401	0.180	0.03
	Ilr_2	-0.282	0.146	0.05
Fully adjusted	Ilr_1	0.383	0.186	0.04
	Ilr_2	-0.369	0.151	0.01
Composition B: Arm elevation >60˚ in upright body position, arm elevation ≤60˚ in upright body position, and total non-upright body position				
Crude	Ilr_1	0.316	0.132	0.02
	Ilr_2	-0.252	0.138	0.07
Fully adjusted	Ilr_1	0.3227	0.138	0.02
	Ilr_2	-0.353	0.145	0.02
Composition C: Arm elevation >90˚ in upright body position, arm elevation ≤90˚ in upright body position, and total non-upright body position				
Crude	Ilr_1	0.304	0.104	0.004
	Ilr_2	-0.266	0.129	0.04
Fully adjusted	Ilr_1	0.310	0.107	0.004
	Ilr_2	-0.361	0.134	0.007

Crude: models adjusted for age and sex, fully adjusted: models adjusted for age, sex, body mass index, work time spent with lifting/carrying, and type of work (blue-collar or white-collar).

Appendix C. Results of the prediction method to interpret ilr-based effect sizes obtained from Cox Proportional Hazard Models in terms of change in LTSA risk corresponding to the theoretical change in minutes of work time composition A (arm elevation >30˚ in upright position, arm elevation ≤30˚ in upright position and total non-upright position), B (arm elevation >60˚ in upright position, arm elevation ≤60˚ in upright position and total non-upright position) and C (in upright position with arm elevation >90˚, in upright position with arm elevation ≤90˚ and total non-upright position)

Number of reallocated composition	Change in composition (mins)	Reallocated composition (mins)			Worktime (mins)	95%CI Low	Hazard ratio	95%CI high
**Composition A**		Arm elevation >30˚	Arm elevation ≤30˚	Non-upright				
			**First Set**					
1	-2.0	92.2	209.8	155	457	0.980	0.989	0.999
2	-1.5	92.7	209.3	155	457	0.985	0.992	0.999
3	-1.0	93.2	208.8	155	457	0.990	0.995	0.999
4	-0.5	93.7	208.3	155	457	0.995	0.997	1.000
**Reference**	**0**	**94.2**	**207.8**	**155**	**457**	**1.000**	**1.000**	**1.000**
6	0.5	94.7	207.3	155	457	1.000	1.003	1.005
7	1.0	95.2	206.8	155	457	1.001	1.005	1.010
8	1.5	95.7	206.3	155	457	1.001	1.008	1.015
9	2.0	96.2	205.8	155	457	1.001	1.011	1.020
			**Second Set**					
**Reference**	**0**	**24.1**	**277.9**	**155**	**457**	**1.000**	**1.000**	**1.000**
1	20	44.1	257.9	155	457	1.018	1.247	1.528
2	40	64.2	237.8	155	457	1.032	1.450	2.036
3	50	74.2	227.8	155	457	1.039	1.545	2.295
4	60	84.2	217.8	155	457	1.047	1.637	2.562
5	70	94.2	207.8	155	457	1.054	1.730	2.840
6	80	104.2	197.8	155	457	1.061	1.823	3.131
7	90	114.2	187.8	155	457	1.068	1.917	3.440
8	100	124.3	177.7	155	457	1.076	2.013	3.769
9	120	144.3	157.7	155	457	1.092	2.218	4.506
10	140	164.3	137.7	155	457	1.109	2.445	5.389
**Composition B**		Arm elevation >60˚	Arm elevation ≤60˚	Non-upright				
			**First Set**					
1	-2.0	14.7	287.3	155	457	0.936	0.964	0.994
2	-1.5	15.2	286.8	155	457	0.952	0.974	0.995
3	-1.0	15.7	286.3	155	457	0.968	0.983	0.997
4	-0.5	16.2	285.8	155	457	0.984	0.991	0.998
**Reference**	**0**	**16.7**	**285.3**	**155**	**457**	**1.000**	**1.000**	**1.000**

6	0.5	17.2	284.8	155	457	1.001	1.008	1.015
7	1.0	17.7	284.3	155	457	1.003	1.017	1.031
8	1.5	18.2	283.8	155	457	1.004	1.025	1.046
9	2.0	18.7	283.3	155	457	1.006	1.033	1.061
			**Second Set**					
**Reference**	**0**	**3**	**299**	**155**	**457**	**1.000**	**1.000**	**1.000**
2	4	7	295	155	457	1.041	1.273	1.558
3	9	12	290	155	457	1.068	1.483	2.059
4	14	17	285	155	457	1.087	1.639	2.472
5	19	22	280	155	457	1.101	1.768	2.837
6	24	27	275	155	457	1.113	1.879	3.172
7	29	32	270	155	457	1.124	1.980	3.487
8	34	37	265	155	457	1.134	2.072	3.788
9	39	42	260	155	457	1.143	2.159	4.078
10	44	47	255	155	457	1.151	2.241	4.362
**Composition C**		Arm elevation >90˚	Arm elevation ≤90˚	Non-upright				
			**First Set**					
1	-2.0	1.0	301.0	155		0.624	0.754	0.912
2	-1.5	1.5	300.5	155		0.742	0.837	0.943
3	-1.0	2.0	300.0	155		0.840	0.901	0.966
4	-0.5	2.5	299.5	155		0.924	0.954	0.985
**Reference**	**0**	**3.0**	**299.0**	**155**		**1.000**	**1.000**	**1.000**
6	0.5	3.5	298.5	155		1.013	1.041	1.069
7	1.0	4.0	298.0	155		1.025	1.077	1.132
8	1.5	4.5	297.5	155		1.035	1.111	1.192
9	2.0	5.0	297.0	155		1.044	1.141	1.247
			**Second Set**					
**Reference**	**0**	**<1**	**302**	**155**		**1.000**	**1.000**	**1.000**
2	2	2	300	155		1.123	1.428	1.815
3	3	3	299	155		1.162	1.585	2.161
4	3	3	299	155		1.162	1.585	2.161
5	4	4	298	155		1.191	1.707	2.447
6	6	6	296	155		1.233	1.897	2.919
7	8	8	294	155		1.264	2.046	3.311
8	10	10	292	155		1.289	2.171	3.654
9	12	12	290	155		1.311	2.279	3.964

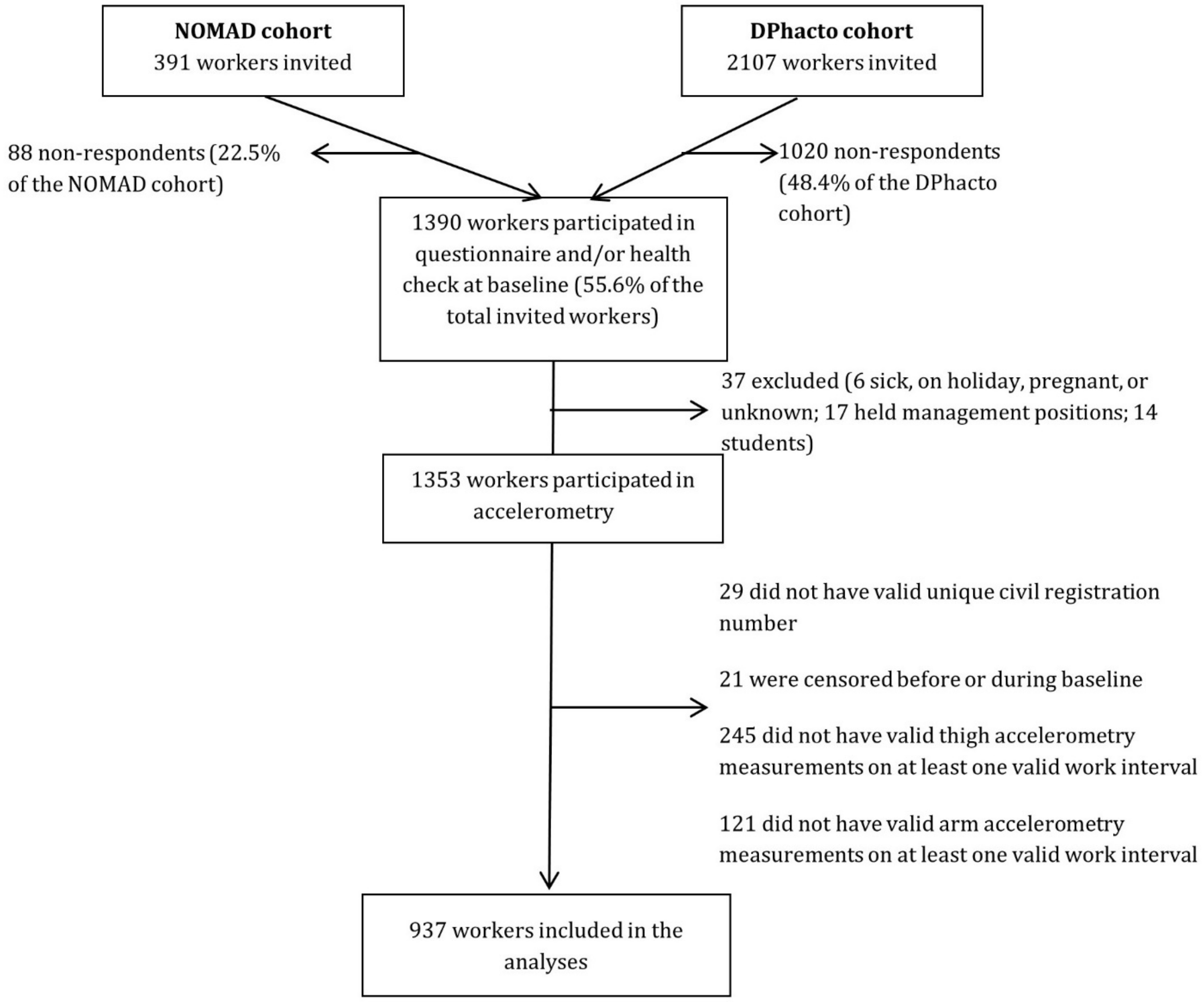
NOMAD cohort
391 workers invited
DPhacto cohort
2107 workers invited
88 non-respondents (22.5% of the NOMAD cohort)
1020 non-respondents (48.4% of the DPhacto cohort)
1390 workers participated in questionnaire and/or health check at baseline (55.6% of the total invited workers)
37 excluded (6 sick, on holiday, pregnant, or unknown; 17 held management positions; 14 students)
1353 workers participated in accelerometry
29 did not have valid unique civil registration number
21 were censored before or during baseline
245 did not have valid thigh accelerometry measurements on at least one valid work interval
121 did not have valid arm accelerometry measurements on at least one valid work interval
937 workers included in the analyses

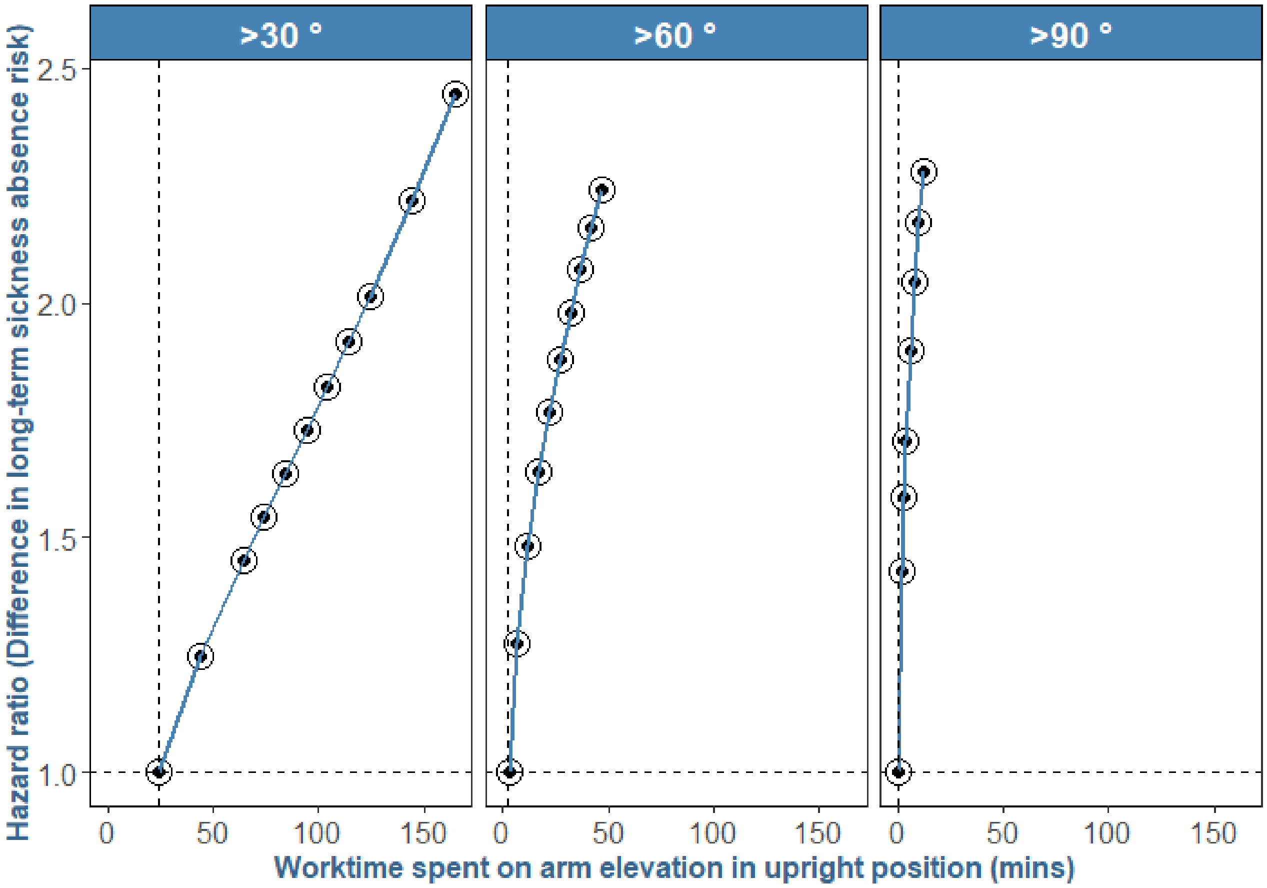

>30 °
>60 °
>90 °
Hazard ratio (Difference in long-term sickness absence risk)
2.5
2.0
1.5
1.0
0
50
100
150
Worktime spent on arm elevation in upright position (mins)

Appendix F. Results of the comparison between the three sensitivity analyses and main analyses investigating the association between the composition of work time spent with arm elevation and risk of long-term sickness absence. These three analyses were (1) analyzing the arm elevation during the whole work time –i.e., both during upright and non-upright body positions- instead of arm elevation in only upright body position, (2) adjusting for the potential confounding effect of influence at work compared to not adjusting for it, and (3) removing workers from the analysis who had a pre-event of LTSA compared to not removing them.

Type of analyses	n	Main analyses	n	Sensitivity analyses
1. analyzing the arm elevation during the whole work time –i.e., both during upright and non-upright body positions- instead of arm elevation in only upright body position	937	Compostion A*: LR Chisq=6.0, P=0.05<br>Composition B:* LR Chisq=6.6, P=0.04<br>Composition C*: LR Chisq=9.9, P=0.007	937	Compostion A**: LR Chisq=9.2, P=0.002<br>Compostion B**: LR Chisq=4.4, P=0.04<br>Compostion C**: LR Chisq=2.1, P=0.15
2. Adjusting/not adjusting for the potential confounding effect of influence at work	733	Compostion A*: LR Chisq=6.0, P=0.05<br>Compostion B*: LR Chisq=6.5, P=0.04<br>Compostion C*: LR Chisq=9.7, P=0.01	733	Compostion A*: LR Chisq=5.5, P=0.06<br>Compostion B*: LR Chisq=5.5, P=0.06<br>CompostionC*: LR Chisq =8.6, P=0.01
3. Removing/not removing workers with pre event of LTSA	937	Compostion A*: LR Chisq=6.0, P=0.05<br>Compostion B*: LR Chisq=6.6, P=0.04<br>Compostion C*: LR Chisq=9.9, P=0.01	880	Compostion A*: LR Chisq=5.3, P=0.07<br>Compostion B*: LR Chisq=6.1, P=0.05<br>Compostion C*: LR Chisq=6.9, P=0.03

LR Chisq= Likelihood ratio chi-square statistics obtained from the cox model; *Composition A = arm elevation >30˚ in upright body position, arm elevation ≤30˚ in upright body position, and non-upright body position (Figure 1A); *Composition B= arm elevation >60˚ in upright body position, arm elevation ≤60˚ in upright body position, and non-upright body position (Figure IB); and *Composition C= arm elevation >90˚ in upright body position, arm elevation ≤90˚ in upright body position, and non-upright body position (Figure IC); ** Composition A = arm elevation >30˚ during whole work time, arm elevation ≤30˚ during the whole worktime; ** Composition B= arm elevation >60˚ during the whole worktime, arm elevation ≤60˚ during the whole worktime, and ** Composition C= arm elevation >90˚ during the whole worktime, arm elevation ≤90˚ during the whole worktime.

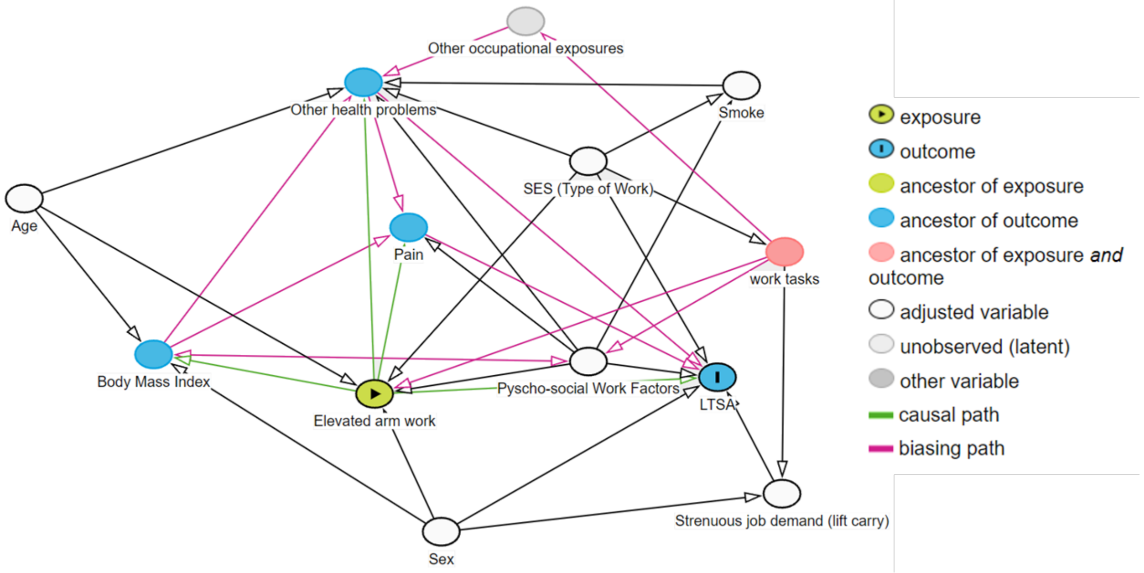

Appendix G. Directed acyclic graph indicating the potential confounders that needed to be adjusted for when investigating the association between worktime spent on elevated arm work and risk of long-term sickness absence (LTSA).

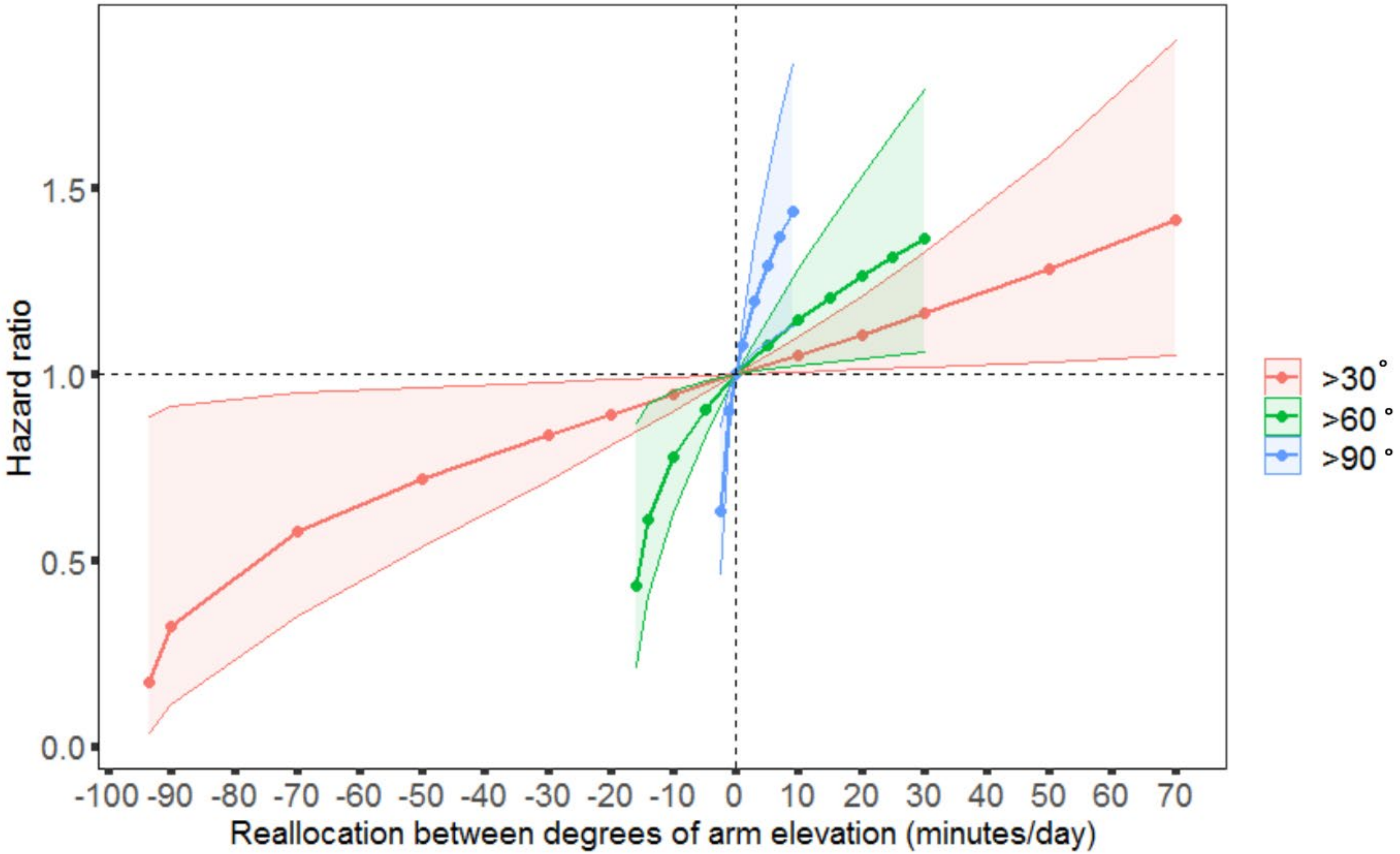

Appendix H. The direction and strength of the association between work time spent with arm elevation >30˚, >60˚ and >90˚ in upright position, relative to work time spent with arm elevation ≤30˚, ≤60˚, and ≤90˚, respectively, and prospective risk of long-term sickness

absence. The X-axis represents the range of reallocations between; composition A: >30˚ and ≤30˚, composition B: >60˚ and ≤60˚, and composition C: >90˚ and ≤90˚; in upright position. Y-axis indicates the ratio of the hazards associated with the new reallocated composition and reference composition (average composition). “0” on y axis represents risk associated with reference average composition. The displayed association looks non-linear for panel “>90˚”. This is because when linear equations are performed on ilrs (the transformed composition A, B or C) and the results are then anti-logged, the results appear to be non-linear (1).

1. Dumuid D, Pedišić Ž, Palarea-Albaladejo J, Martín-Fernández JA, Hron K, Olds T. Compositional Data Analysis in Time-Use Epidemiology: What, Why, How. Int J Environ Res Public Health. 2020 Mar 26;17(7).
